# Evidence and determinants of post-abortion family planning utilization among women of reproductive age in Africa: an umbrella review

**DOI:** 10.3389/frph.2025.1687886

**Published:** 2025-11-13

**Authors:** Doreen Kainyu Kaura, Dereje Bayissa Demissie, Stefan Gebhardt

**Affiliations:** 1 School of Nursing, Faculty of Community and Health Sciences, University of the Western Cape, Cape Town, South Africa; 2 Postdoctoral research Fellow at Faculty of Medicine and Health Sciences, Stellenbosch University, Cape Town, South Africa; 3 Department of Obstetrics and Gynecology, Faculty of Medicine and Health Sciences, Stellenbosch University, Cape Town, South Africa

**Keywords:** Africa, determinants, global health policy, post-abortion family planning utilization, prevalence, umbrella review, women of reproductive aget

## Abstract

**Background:**

Post-abortion family planning (PAFP), ideally initiated within 48 h, is crucial for preventing unplanned pregnancies. Repeat-induced abortion remains a significant challenge to the sexual and reproductive health of women. Despite numerous systematic reviews and meta-analyses, inconsistent findings still hinder effective policy formulation and clinical decision-making. In Africa, high rates of unsafe abortion and unintended pregnancy persist, exacerbated by socioeconomic and health system barriers. This umbrella review consolidates global evidence on the prevalence, determinants, and use of PAFP to inform health policy, strengthen service delivery, and promote reproductive health equity, especially in regions with limited access to safe abortion services and contraception.

**Methods:**

An umbrella review of systematic reviews and meta-analyses based on observational studies was conducted. The methodological quality of the included studies was assessed using the Assessment of Multiple Systematic Reviews tool. Heterogeneity was assessed using Cochran's Q and *I*^2^ statistics, while publication bias was evaluated using Egger's test and funnel plots. A random-effects meta-analysis was used to estimate the pooled effect size, with analyses performed using Stata version 19. Subgroup analyses were performed by country and continent. Pooled results were synthesized using random-effects meta-analysis models. The review protocol was registered with PROSPERO (CRD420251089314).

**Result:**

This umbrella review included six systematic reviews and meta-analyses, comprising 198 primary studies conducted across 44 African countries, with a combined sample size of 420,832 women of reproductive age assessing post-abortion family planning utilization. This umbrella review found that the pooled prevalence of post-abortion family planning utilization among women of reproductive age in Africa was 62.82% (95% CI: 59.24%–66.40%), indicating substantial uptake but with high heterogeneity across studies (*I*^2^ = 98.81%). A subgroup umbrella meta-analysis revealed that the pooled prevalence of post-abortion family planning utilization was 69.31% (95% CI: 64.27%–74.35%) in Ethiopia, compared to 60.29% (95% CI: 57.11%–63.47%) in other African countries. This study determined that injectables were the most commonly utilized post-abortion family planning method (34.12%), followed by pills and implants, each with a nearly equal share of 22%. This umbrella review identified key pooled determinants of post-abortion family planning utilization among women of reproductive age in Africa, including marital status (being married), younger maternal age (15–24 years), educational attainment, receipt of post-abortion family planning counseling, prior use of family planning, history of abortion, unintended pregnancy, and contraceptive knowledge.

**Conclusion and recommendation:**

The high prevalence of PAFP utilization in Africa (62.82%), and particularly in Ethiopia (69.31%), reflects encouraging progress. However, the fact that 37.18% of post-abortion women in Africa and 30.69% in Ethiopia still do not use PAFP underscores a critical gap that demands targeted policy action. The predominance of injectable contraceptives highlights the need to expand access to a broader range of methods, including long-acting reversible contraceptives, to support informed and voluntary choice. Policymakers and health planners in Africa should urgently strengthen reproductive health policies by implementing a comprehensive, multipronged strategy to ensure universal access to post-abortion care.

**Systematic Review Registration:**

https://www.crd.york.ac.uk/PROSPERO/view/CRD420251089314, PROSPERO CRD420251089314.

## Introduction

According to a 2023 report by the World Health Organization (WHO), over 700 women died daily that year from preventable pregnancy and childbirth complications. Approximately 75% of maternal deaths were due to severe bleeding, infections, high blood pressure, delivery complications, and unsafe abortions, most of which are treatable (1, 2). The WHO defines abortion as the termination of a pregnancy before 20 weeks of gestation or when the fetus weighs less than 500 g. Abortion may occur spontaneously (miscarriage) or be induced. Safe abortions are those that follow WHO-recommended methods appropriate for the duration of pregnancy and are performed by trained providers using either medication or simple outpatient procedures (3, 4). Abortion is a complex issue influenced by religious, moral, cultural, and political factors and remains a significant public health concern. Over a quarter of the global population lives under restrictive abortion laws. Despite legal status, abortions continue to occur and nearly half are unsafe, performed by unskilled providers or in unhygienic conditions (5).

A study by Bell et al. on the global epidemiology of induced abortion found that over 73 million abortions occur annually worldwide. While abortion rates are declining in high-resource settings, they remain stable in low- and middle-resource countries, where unsafe abortions persist as a major cause of maternal mortality (6).

According to the 2023 Comprehensive Abortion Care (CAC) report by the WHO, maternal mortality in Southeast Asia decreased by 57.3% between 2007 and 2017; however, unsafe abortion remains a significant concern. CAC, which includes post-abortion family planning, plays a crucial role in improving maternal health. Limited data collection continues to hinder accurate assessment of service accessibility and quality (7).

The 2022 WHO Abortion Care Guideline emphasizes the importance of safe, accessible services, including self-managed medical abortion and expanded provider roles. These updated guidelines strengthen post-abortion family planning by promoting autonomy, community-based care, and improved access to abortion pills; these measures are key to reducing repeat unintended pregnancies and enhancing reproductive health through evidence-based, rights-centered service delivery across diverse legal and clinical settings (8).

According to Kim et al., CAC provides safe, accessible, and woman-centered services encompassing pre-abortion, abortion, and post-abortion care. CAC helps reduce maternal mortality and can be delivered by various healthcare providers. However, barriers such as restrictive laws and provider beliefs persist. A holistic, rights-based approach is essential to improving health outcomes and promoting gender equality, especially in humanitarian settings (9). The 2022 WHO Abortion Care Guideline frames access to safe abortion, including post-abortion family planning, as a health and human rights priority. It promotes immediate initiation of contraception after abortion and expands provider roles to include community health workers and pharmacists (9, 10).

Regarding post-abortion care, a previous scoping review by Macleod et al. reported that non-barrier contraceptive use ranged from 15% to 76%, unintended pregnancy from 24% to 91%, and abortion from 11% to 48% (11). A systematic review of randomized controlled trials identified that the key contributing factors included alcohol use, violence, health system failures, socioeconomic challenges, and pregnancy desire linked to non-paying partners (12). Kim et al., along with the World Health Organization, emphasized that comprehensive abortion care is essential for safeguarding sexual and reproductive health (SRH), saving lives, and preserving dignity. The WHO guideline promotes accessible, evidence-based care to ensure safe, effective, and equitable services (9, 10).

Sub-Saharan Africa accounts for 29% of global unsafe abortions and 62% of abortion-related deaths, largely due to restrictive laws, poor post-abortion care, and limited access to contraception (13). Another study by Woldemichael et al. underscored the critical role of post-abortion family planning (PAFP) in reducing unintended pregnancies and unsafe abortions in Eastern Africa. Utilization rates of PAFP range from 53.7% to 67.86% and are influenced by factors such as education, counseling, and marital status. Injectables and implants were identified as the most preferred methods (14, 15). Despite its benefits, nearly half of women in some areas still do not utilize PAFP, highlighting the need for improved counseling and awareness.

Previous systematic reviews and meta-analyses have reported inconsistent prevalence rates of post-abortion family planning (PAFP) utilization in Eastern Africa and Ethiopia, ranging from 67.86% to 74.56%. Factors influencing uptake include counseling, prior contraceptive use, marital status, and education, with injectables, implants, and pills identified as the most preferred methods (15–18). Although PAFP utilization remains below WHO targets, it shows promise in reducing unintended pregnancies and repeat abortions (9, 10).

However, existing reviews differ in scope, methodology, and regional focus, resulting in fragmented and contradictory findings that hinder effective policy formulation and clinical decision-making. Many prior reviews lack a comprehensive synthesis and overlook contextual factors affecting PAFP uptake. This umbrella review aims to consolidate evidence from published systematic reviews and meta-analyses to provide a clearer understanding of the prevalence and determinants of PAFP among women of reproductive age in Africa. The findings will inform health policy, improve service delivery, and promote reproductive health equity in low-resource settings.

## Review objectives

1. To synthesize evidence from systematic reviews and meta-analyses on the prevalence of post-abortion family planning utilization among women in Africa;
2. To identify pooled predictors of post-abortion family planning utilization among women in Africa; and
3. To evaluate the effectiveness of post-abortion care interventions among women of reproductive age in Africa.

## Methods

This umbrella review was conducted following established methodologies for synthesizing multiple systematic reviews (19). The procedure adheres to the umbrella review methodology developed by the Joanna Briggs Institute (20). It involved a systematic synthesis of eligible systematic reviews and meta-analyses (SRMs) summarizing the global prevalence, determinants, and post-abortion care interventions related to pregnancy termination.

This umbrella review was conducted in accordance with the Preferred Reporting Items for Systematic Reviews and Meta-Analysis (PRISMA) guidelines and was registered in PROSPERO under reference number CRD420251089314.

### Operational definitions

Umbrella Review: An umbrella review is a systematic synthesis of multiple systematic reviews and meta-analyses on a specific topic. It provides a high-level overview of existing evidence; identifies patterns, gaps, and inconsistencies; and offers comprehensive insights to inform policy and practice (19).

Post-Abortion Family Planning (PAFP): PAFP refers to the provision and uptake of contraceptive services immediately following an abortion. It aims to prevent unintended pregnancies and repeat abortions by offering timely, informed, and voluntary access to family planning methods (9, 10).

Determinants: In the context of this review, determinants are factors that influence the utilization of post-abortion family planning services. These may include sociodemographic characteristics (e.g., age, marital status, and education), prior contraceptive use, receiving counseling, and access to healthcare services (15–18).

### Search strategy

Six major international online databases—PubMed, Scopus, Science Direct, Web of Science, and databases specific to systematic reviews and meta-analyses such as the Cochrane Database of Systematic Reviews and the Database of Abstracts of Reviews of Effects—were searched for systematic reviews and meta-analyses (SRMs) examining the prevalence, determinants, and utilization of post-abortion family planning among women of reproductive age globally. A comprehensive search strategy was employed using adapted PICO questions, developed from relevant keywords and Medical Subject Headings (MeSH) terms. These search terms were combined using Boolean operators “OR” and “AND” to ensure a thorough and inclusive search.

(a) Population: females (adolescents, youths, and women of reproductive age) who have undergone an abortion.
(b) Intervention: utilization of any post-abortion family planning service or method (counseling, method availability, etc.).
(c) Comparison: women of reproductive age post-abortion who were not offered post-abortion family planning services.
(d) Outcome: prevalence, determinants, and uptake of post-abortion family planning.
(e) Study design: systematic reviews and meta-analyses of observational studies.
(f) Setting (context): Africa.

Both published and unpublished studies were included in this umbrella review.

A comprehensive literature search was conducted between 12 June and 1 July 2025, without restrictions on publication date, and included all relevant studies published up to 1 July 2025. Two independent researchers conducted the search, and any discrepancies were resolved through discussion and consensus with the remaining authors.

A sample search strategy for PubMed was developed using a combination of Medical Subject Headings (MeSH) terms and free-text keywords. The PubMed search string included terms such as ((((((Abortion OR “Abortion, Induced”/trends[Mesh] OR Prevalence OR Epidemiology OR Magnitude) AND (determinants OR associated factors OR predictors) AND post-abortion contraceptive uptake OR (“Contraceptive Agents”, OR (family planning use OR family planning utilization OR family planning uptake OR family planning services OR contraceptive use OR contraceptive utilization OR contraceptive uptake OR birth control OR fertility control OR population control) AND (systematic review and meta-analysis)). The complete search strategy details are provided in Supplementary File 1: Search strategy.docx.

### Inclusion criteria

This umbrella review included all available systematic reviews and meta-analyses (SRMs) that met predefined eligibility criteria: a clearly defined literature search strategy, quality appraisal of included studies using appropriate tools, and a standardized approach to pooling data and summarizing estimates among women of reproductive age. Both published and unpublished studies were considered, with inclusion limited to those written in English. A comprehensive literature search was conducted between 12 June and 1 July 2025, covering studies published from 1 January 2000 to 1 July 2025. This broad inclusion period aimed to capture diverse evidence across time and regions, ensuring a robust synthesis of findings related to post-abortion family planning (PAFP) utilization in Africa.

### Exclusion criteria

Studies were excluded if they did not report on the prevalence or determinants of post-abortion family planning (PAFP) utilization among women of reproductive age. Qualitative reviews, narrative reviews, editorials, correspondence, abstracts, and methodological papers were also excluded. Additionally, literature reviews lacking a clearly defined research question, search strategy, or article selection process were not considered. This rigorous screening process ensured the inclusion of high-quality and relevant evidence to support a comprehensive synthesis of PAFP utilization in Africa.

### Data extraction

Data from the included SRM studies were extracted using a standardized Excel form. The extracted information included study identification; review objectives; prevalence and risk factors of induced, repeat, and unsafe abortions; associated outcomes; and post-abortion care. Additional data covered effect sizes (OR/RR with 95% CI), the number and design of primary studies, sample sizes, methods and scores used for publication bias and quality assessment, data synthesis models, and key conclusions. Full details of the extraction process are provided in Supplementary Files 3–3.2.

### Risk of bias assessment

All included studies were critically appraised to assess the validity and scoring of their findings. This umbrella review used the Assessment of Multiple Systematic Reviews (AMSTAR-2) tool to ensure the methodological and evidence quality of the included SRM studies (19, 21) (details are provided in Supplementary File 2: AMSTAR-2 .xlsx).

### Data synthesis

Both narrative (qualitative) and quantitative approaches were used to summarize the findings of the included SRM studies. When multiple estimates were reported for the magnitude, associated factors, or adverse outcomes of pregnancy termination (including induced, repeat, and unsafe abortions among reproductive-age women), the range of estimates was presented, and a pooled summary estimate was calculated. The choice of meta-analysis model was guided by the level of heterogeneity, assessed using Higgins' *I*^2^ statistic (22). Due to the anticipated high between-study heterogeneity, the DerSimonian–Laird random-effects model was employed (22).

Publication bias was assessed if at least 10 studies were included, as this is the minimum typically required for such evaluation (19, 23). The meta-analysis involved calculating pooled estimates of sensitivity, specificity, positive predictive value, and true positives, either directly reported or derived from the included studies. Heterogeneity was evaluated using both Cochran's *Q* test and the *I*^2^ statistic (24). An *I*^2^ value greater than 50% and a Cochran's *Q* test *p*-value <0.05 were considered indicative of significant heterogeneity, warranting the use of a random-effects model (24).

Pooled estimates were calculated using STATA version 17, employing the “metaprop” command with sample size as the weighting variable and 95% confidence intervals. Quantitative analyses were conducted using STATA version 17.0. A summary of predictors of pregnancy termination and post-abortion care utilization, along with their respective odds ratios, was computed.

### Ethical consideration

This study did not require ethical approval or informed consent from participants, as it utilized data solely extracted from previously published SRM studies.

## Results

### Study screening and selection

**Figure 1:**
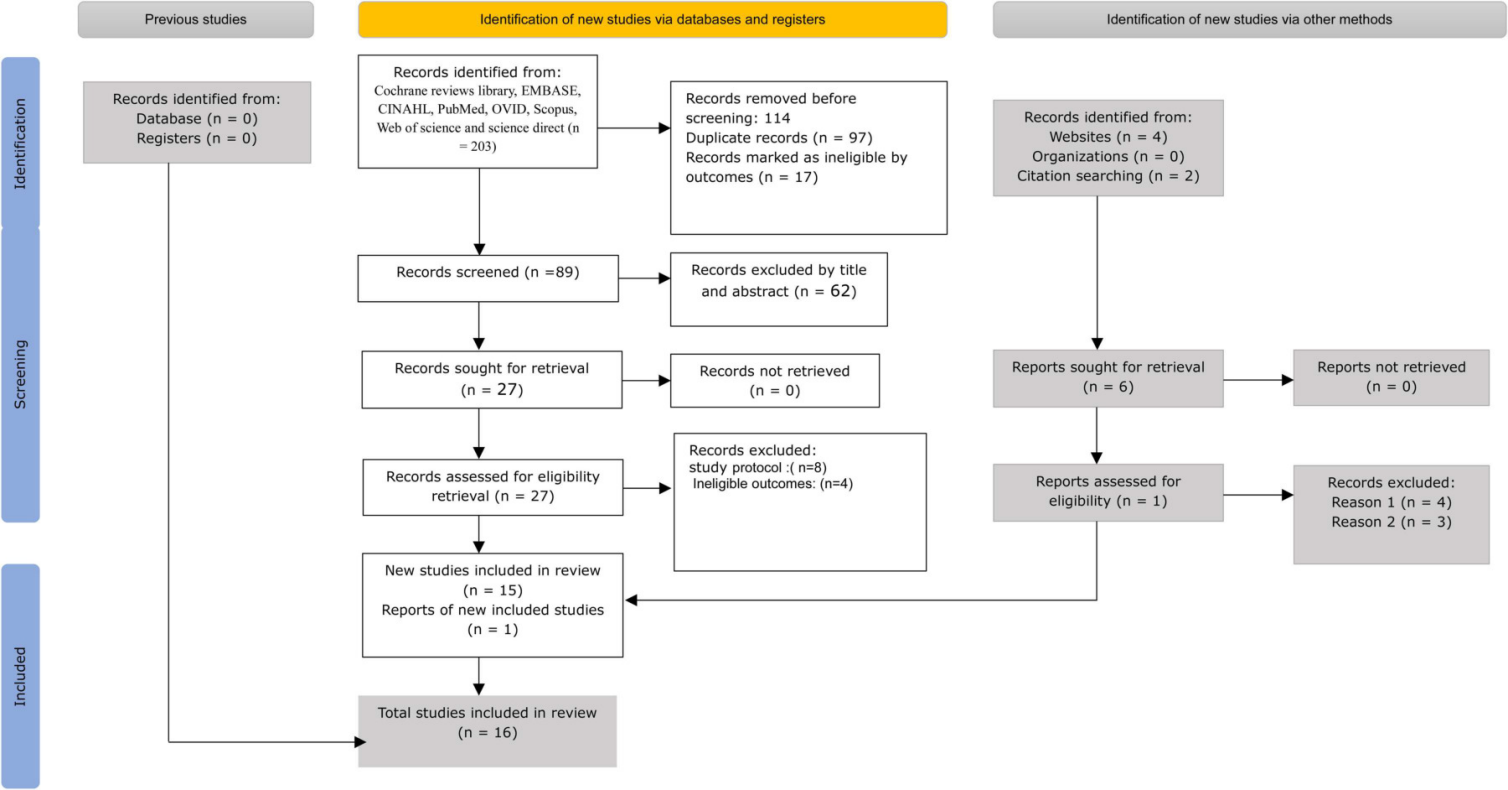
PRISMA 2020 flow diagram for new systematic reviews, which include searches of databases, registers, and other sources (37) from 2000 to 2025.

Two reviewers independently screened the titles and abstracts of 89 systematic reviews and meta-analyses (Supplementary File 1: Search strategy). From this initial screening, 27 articles were selected for full-text review. An additional six articles were identified through reference lists and other databases, resulting in a total of 33 articles undergoing full-text assessment. Of these, 16 met the inclusion criteria for this umbrella review of systematic reviews and meta-analyses (12, 15, 16, 18, 25–36). The number of studies identified, included, and excluded at each stage of the selection process is illustrated in the PRISMA flowchart (Figure 1). The primary reasons for exclusion at the full-text review stage, as indicated in the PRISMA diagram, were ineligible outcomes and study protocols.

### Highlights of conclusions by authors across systematic reviews and meta-analyses of post-abortion family planning utilization

This umbrella review synthesizes findings from multiple systematic reviews and meta-analyses focused on post-abortion family planning utilization in Africa. Bizuneh and Azeze identified key determinants of PAFP uptake in Eastern Africa, including marital status, multiparty, and history of abortion, receipt of counseling, and prior contraceptive use (15). Dagnew and Asresie reported that post-abortion care uptake across Africa stands at 58.78%, falling short of WHO recommendations. A notable decline in uptake was observed between 2020 and 2023, with injectable contraceptives being the most commonly used method. The authors emphasized the need for improved education, awareness, and client-centered counseling (18). Mengistie et al. found that post-abortion contraceptive use in sub-Saharan Africa remains low. They advocated for scaling up utilization through enhanced educational attainment, stronger advocacy for contraceptive options, and comprehensive post-abortion counseling services (26).

This umbrella review synthesizes findings from multiple systematic reviews and meta-analyses focused on post-abortion family planning utilization in Africa. Beyene et al. reported that PAFP utilization remains suboptimal, with key influencing factors including marital status, educational level, receipt of counseling, prior contraceptive use, and age. These insights are critical for informing reproductive health policy and service delivery (16). Similarly, Wake et al. found that the pooled prevalence of post-abortion contraceptive use in Ethiopia was low, with significant associations observed for counseling and prior use of modern contraceptives (27). Cherie et al. also concluded that family planning utilization among women receiving abortion services in Ethiopia falls below both WHO and national standards (25). Receipt of counseling and a history of contraceptive use were identified as strong predictors of uptake. Collectively, these findings highlight the need for strengthened counseling services and targeted interventions to improve post-abortion contraceptive use in Ethiopia (16, 25, 27). Table 1 summarizes the total number of included studies, study settings, number of countries represented, overall sample sizes, and key conclusions reported by the authors.

**Table 1:** Summary of the total number of included studies, study settings, number of countries represented, overall sample size, and key conclusions reported by the authors, 2013–2025.

Author (reference)	Review aim	No of included studies	Study setting	Total countries	Sample size	Authors' conclusion	Quality MSTAR-2
Beyene et al. (16)	To review post-abortion contraceptive utilization and its factors in Ethiopia	11	Ethiopia	1	4,336	This systematic review found that post-abortion contraceptive utilization in Ethiopia remains suboptimal. Factors significantly associated with utilization included marital status, educational level, receipt of counseling, prior contraceptive exposure, and age. These findings may provide valuable insights for healthcare policymakers aiming to improve reproductive health services	14
Bizuneh and Azeze, (15)	To assess post-abortion family planning utilization and its determinant factors in Eastern Africa	29	Eastern Africa	8	70,037	This systematic review revealed that marital status, multiparty, history of abortion, receiving counseling on post-abortion family planning, and prior contraceptive use were significantly associated with post-abortion family planning utilization	14
Wake et al. (27)	To estimate the pooled prevalence of post-abortion contraceptive utilization and its associated factors in Ethiopia	14	Ethiopia	1	5,719	This systematic review revealed that the pooled prevalence of post-abortion contraceptive utilization remains low. Significant factors associated with utilization included receiving post-abortion family planning counseling and a history of using modern contraceptive methods	15
Cherie et al. (25)	To estimate the pooled prevalence and associated factors of family planning utilization among women receiving abortion services in Ethiopia	16	Ethiopia	1	8,236	This systematic review revealed that family planning utilization among women receiving abortion services in Ethiopia is below both the WHO and national recommendations. Counseling and a history of contraceptive use were found to be significantly associated with post-abortion family planning utilization	16
Dagnew and Asresie (18)	To assess the uptake of post-abortion contraceptive (PAC) and associated factors among African women who received abortion services	48	African	17	84,205	This systematic review found that the uptake of post-abortion care (PAC) in Africa is 58.78%, which falls short of the WHO's recommendation that all women delay conception for at least 6 months following an abortion. Notably, there was a 20.22% decline in uptake between 2020 and 2023 compared to the 2015–2019 period. To address this gap, it is essential to improve women's awareness of post-abortion contraception, educate them on the risks of early conception, and strengthen both client-centered counseling and women's education	16
						The most widely used contraceptive methods were injectables (30.27%), followed by implants (25.13%), oral contraceptive pills (22.34%), and IUDs (10.47%). This systematic review and meta-analysis found that the pooled prevalence of post-abortion contraceptive use in sub-Saharan Africa remains low. Therefore, the development and implementation of effective strategies are essential to increase uptake. These strategies should include enhancing educational attainment, promoting awareness of post-abortion contraceptive options, and providing comprehensive post-abortion family planning counseling	
Mengistie et al. (26)	To investigate the prevalence and determinant factors of post-abortion family planning use in the region.	80	Sub-Saharan Africa	16	248,299	This meta-analysis indicates that the pooled prevalence of post-abortion contraception use in sub-Saharan Africa remains low. Therefore, appropriate planning and implementation of effective strategies are crucial to scaling up post-abortion family planning use, including improving educational attainment, advocating for post-abortion contraceptive methods, and providing effective post-abortion family planning counseling	16

Assessment of Multiple Systematic Reviews (AMSTAR-2) tool.

### Post-abortion family planning utilization-related characteristics

This umbrella review synthesizes findings from multiple systematic reviews and meta-analyses focused on post-abortion family planning utilization across various African regions. The studies included in the review, published between 2021 and 2025, collectively provide insights into the prevalence, determinants, and regional disparities in post-abortion family planning (PAFP) uptake. Post-abortion family planning reviews mainly focus on Ethiopia and broader African regions, including Eastern, Western, Northern, Central, Southern, and sub-Saharan Africa (15, 16, 18, 25–27). The number of studies included per review ranged from 2 to 80, with sample sizes between 274 (18) and 248,299 (26). Utilization rates varied across regions, with Ethiopia recording the highest at 74.56% (16) and Western Africa recording the lowest at 51.48%. Moderate uptake was observed in Southern (62.23%), Central (60.96%), and Northern Africa (58.78%) (18). All studies underwent clear quality assessments, with consistent AMSTAR-2 ratings between 14 and 16, ensuring methodological rigor and reliability across findings. Detailed results are presented in Table 2.

**Table 2:** Characteristics of included systematic reviews and meta-analyses on post-abortion family planning utilization, 2021–2025.

Author (reference)	Review aim	Search strategy	Total no. of included studies	Study setting	Total countries	Sample size	Risk of bias	Post- abortion FP	SE	Quality AMSTAR-2
Beyene et al. (16)	To review post-abortion contraceptive utilization and its factors in Ethiopia	Google Scholar, PubMed, EMBASE, Scopus, Web of Science, and gray literature databases	11	Ethiopia	1	4,336	The quality appraisal of the included studies was clearly reported	74.56	0.66140	14
Bizuneh and Azeze (15)	To assess post-abortion family planning utilization and its determinant factors in Eastern Africa	Scopus, HINARI, PubMed, Google Scholar, Web of Science electronic databases, and gray literature repository	29	Eastern Africa	8	70,037	The quality appraisal of the included studies was clearly reported	67.86	0.17647	14
Wake et al. (27)	To estimate the pooled prevalence of post-abortion contraceptive utilization and its associated factors in Ethiopia	PubMed, Google Scholar, Science Direct, Cochrane Library, Scopus, CINAHL, Web of Science, and additional searches by using direct Google search, libraries, and preprints	14	Ethiopia	1	5,719	The quality appraisal of the included studies was clearly reported	63.64	0.63609	15
Cherie et al. (25)	To estimate the pooled prevalence and associated factors of family planning utilization among women receiving abortion services in Ethiopia	PubMed, Google Scholar, Science Direct, HINARI and Cochrane Library, and Google	16	Ethiopia	1	8,236	The quality appraisal of the included studies was clearly reported	69.73	0.50624	16
Dagnew and Asresie (18)	To assess the uptake of post-abortion contraceptive (PAC) and associated factors among African women who received abortion services	Databases, including PubMed, Medline, Research Gate, Africa Journal Online, and Europe PMC, were used to retrieve relevant findings on post-abortion contraceptive uptake	48	African	17	84,205	The quality appraisal of the included studies was clearly reported	58.78	0.16963	16
Dagnew and Asresie (18)	To assess the uptake of post-abortion contraceptive (PAC) and associated factors among East African women who received abortion services	Databases, including PubMed, Medline, Research Gate, Africa Journal Online, and Europe PMC, were used to retrieve relevant findings on post-abortion contraceptive uptake	34	East Africa		30,801	The quality appraisal of the included studies was clearly reported	61.3	0.27753	16
Dagnew and Asresie (18)	To assess the uptake of post-abortion contraceptive (PAC) and associated factors among West African women who received abortion services	Databases, including PubMed, Medline, Research Gate, Africa Journal Online, and Europe PMC, were used to retrieve relevant findings on post-abortion contraceptive uptake	9	West Africa		40,446	The quality appraisal of the included studies was clearly reported	51.48	0.24851	16
Dagnew and Asresie (18)	To assess the uptake of post-abortion contraceptive (PAC) and associated factors among North African women who received abortion services	Databases, including PubMed, Medline, Research Gate, Africa Journal Online, and Europe PMC, were used to retrieve relevant findings on post-abortion contraceptive uptake	1	North Africa		276	The quality appraisal of the included studies was clearly reported	58.78	2.96288	16
Dagnew and Asresie (18)		Databases, including PubMed, Medline, Research Gate, Africa Journal Online, and Europe PMC, were used to retrieve relevant findings on post-abortion contraceptive uptake	2	Central Africa		274	The quality appraisal of the included studies was clearly reported	60.96	2.94715	16
Dagnew and Asresie (18)		Databases, including PubMed, Medline, Research Gate, Africa Journal Online, and Europe PMC, were used to retrieve relevant findings on post-abortion contraceptive uptake	2	Southern Africa		12,408	The quality appraisal of the included studies was clearly reported	62.23	0.43523	16
Mengistie et al. (26)	To investigate the prevalence and determinant factors of post-abortion family planning use in the region	PubMed, Research4Life, Scopus, EMBASE, and Google Scholar	80	Sub-Saharan Africa	16	2,48,299	The quality appraisal of the included studies was clearly reported	60.67	0.09803	16

Assessment of Multiple Systematic Reviews (AMSTAR-2) tool.

### Pooled prevalence of post-abortion family planning utilization in Africa

This umbrella review included six systematic reviews and meta-analyses comprising 198 primary studies conducted across 44 African countries, with a combined sample size of 420,832 women of reproductive age assessing post-abortion family planning utilization (15, 16, 18, 25–27). Further details on PAFP utilization are provided in Supplementary File 3.

**Figure 2:**
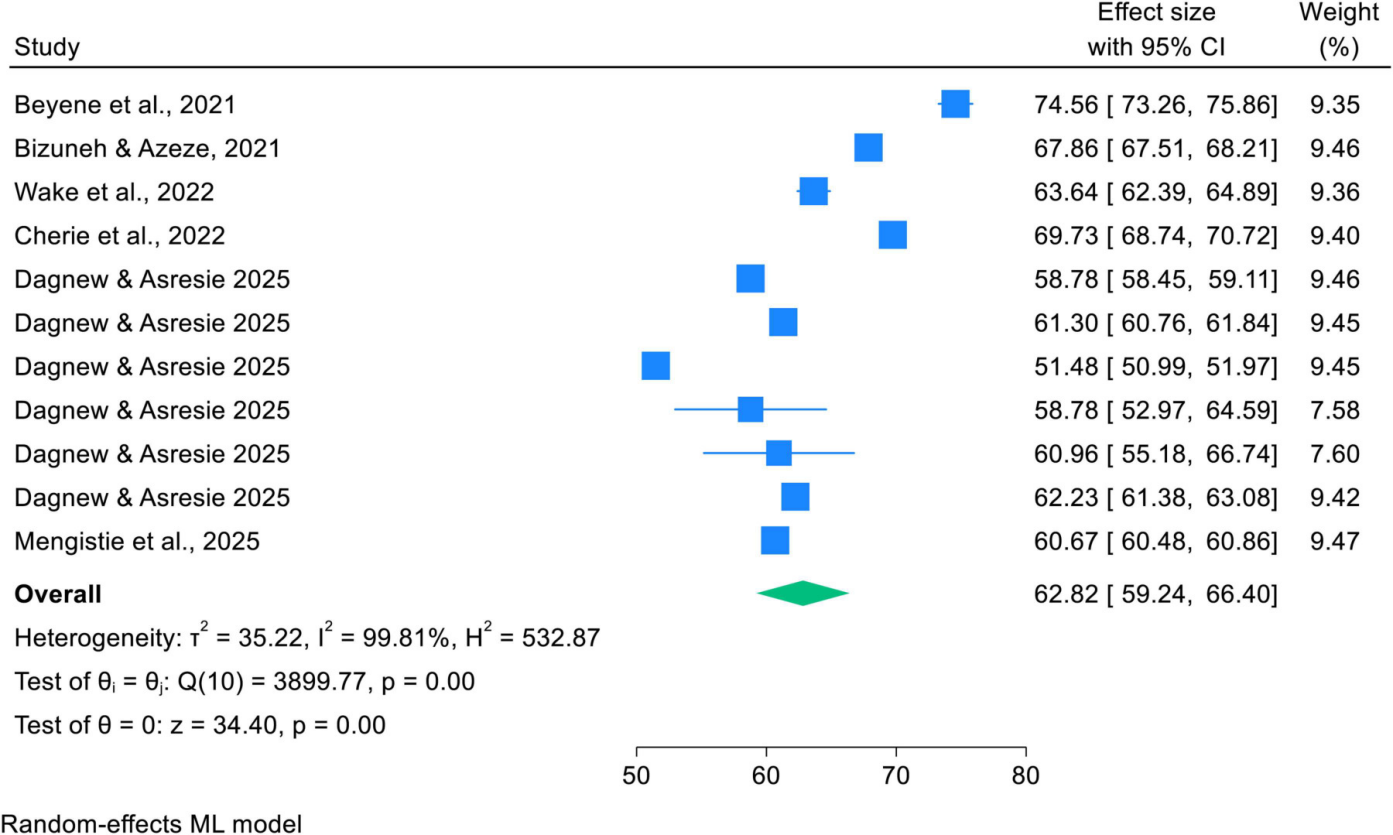
Forest plot showing pooled prevalence of post-abortion family planning utilization among women of reproductive age in Africa in 2025.

This umbrella review of systematic reviews and meta-analyses found that the pooled prevalence of post-abortion family planning utilization among women of reproductive age in Africa was 62.82% (95% CI: 59.24%–66.40%), indicating substantial uptake but with high heterogeneity across studies (*I*^2^ = 98.81%). Detailed results are provided in Figure 2.

### Subgroup analysis by country

**Figure 3:**
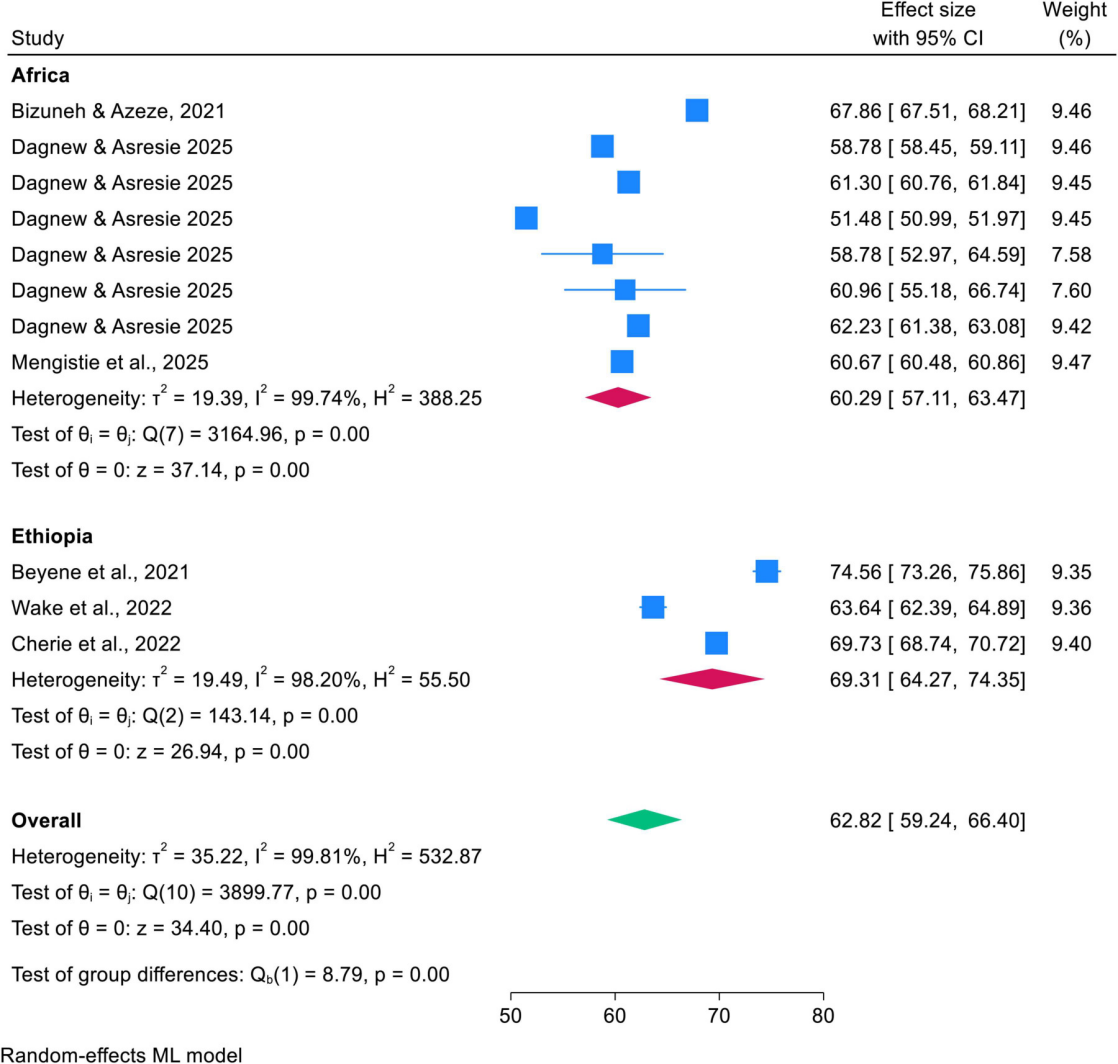
Forest plot showing the subgroup analysis by country for pooled prevalence of post-abortion family planning utilization among women of reproductive age in Africa in 2025.

A subgroup umbrella meta-analysis revealed that the pooled prevalence of post-abortion family planning utilization was 69.31% (95% CI: 64.27%–74.35%) in Ethiopia (16, 25, 27), compared to 60.29% (95% CI: 57.11%–63.47%) in other African countries (15, 18, 26), indicating substantial heterogeneity (*I*^2^ = 98.20%). Detailed results are provided in Figure 3.

**Figure 4:**
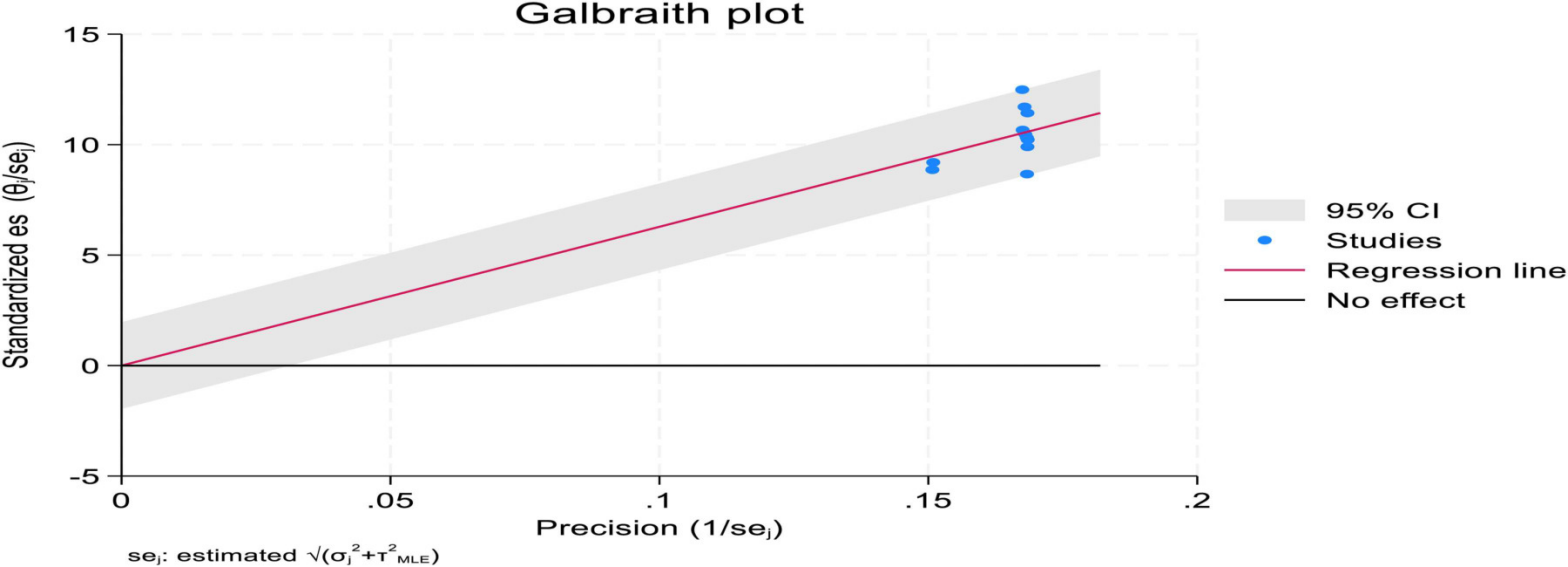
Galbraith plot showing high heterogeneity among included studies on post-abortion family planning utilization among women of reproductive age in Africa in 2025.

The Galbraith plot showed high heterogeneity, with no studies falling outside the 95% confidence band. The symmetry around the red regression line suggests low publication bias. However, the extensive spread of points confirms substantial variability, supporting the observed *I*^2^ = 99.81% heterogeneity. Further details are shown in Figure 4.

### Publication bias assessment

**Figure 5:**
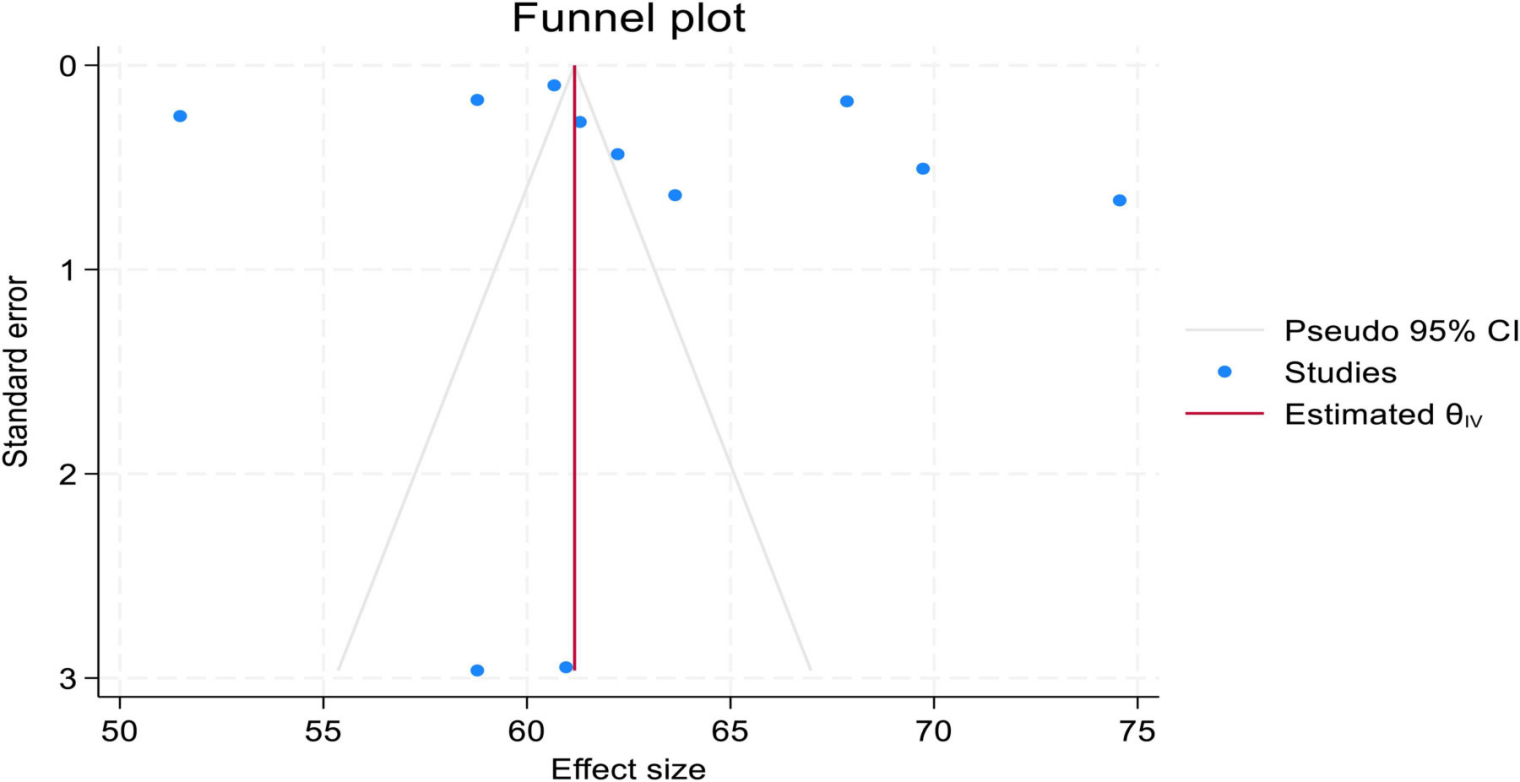
Funnel plot showing asymmetric distribution of included studies in pooled prevalence of post-abortion family planning utilization among women of reproductive age in Africa in 2025.

Publication bias was assessed using a funnel plot, which appeared asymmetric and skewed to the right. However, Egger's regression test (PV = 0.7517) confirmed the absence of significant publication bias (Figure 5).

**Figure 6:**
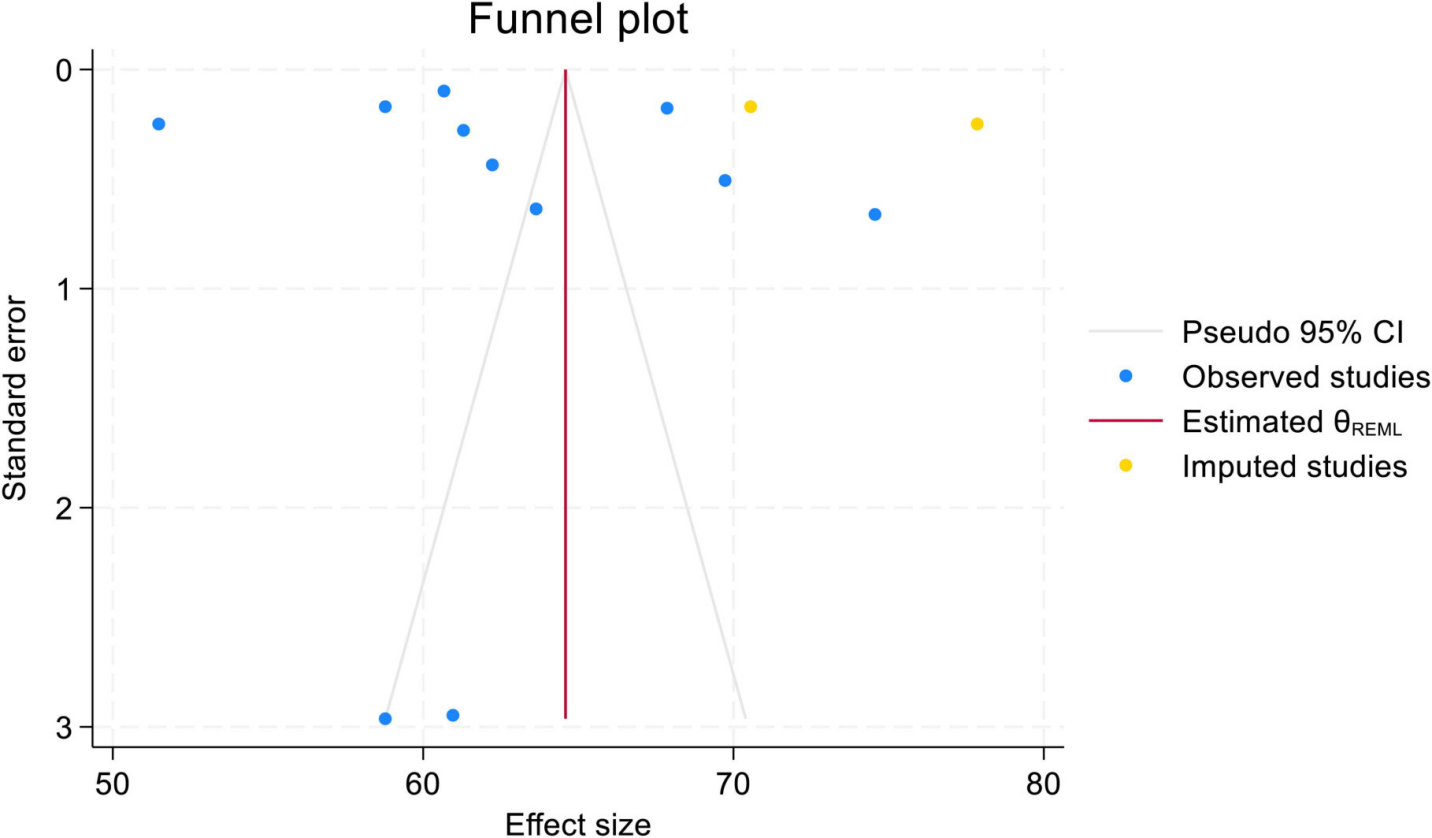
Funnel plot showing symmetric distribution of included studies in pooled prevalence of post-abortion family planning utilization among women of reproductive age in Africa in 2025.

Based on the trim-and-fill analysis, the linear estimator imputed three studies on the right (11 + 2 imputed = 13 studies). The pooled African prevalence of post-abortion family planning utilization among women of reproductive age increased from 62.810% (59.062%–66.558%) to 64.589% (60.591%–68.587%) after the trim-and-fill analysis. Both have narrow confidence intervals, suggesting high precision. This implies that the true effect size might be underestimated when considering only the observed studies. As shown in Figure 5, the funnel plots appeared symmetric after the trim-and-fill analysis, with the linear estimator imputing two studies on the right (Figure 6).

### Common types of post-abortion family planning utilization (PAFP)

This study determined that, among family planning methods used during post-abortion care in Africa, injectables were the most commonly utilized (34.12%), followed by pills and implants, each with a nearly equal share of 22% (15, 26). Further details on types of methods used are provided in Supplementary File 3.1 and Table 3.

**Table 3:** Common methods of post-abortion family planning utilization in Africa in 2025.

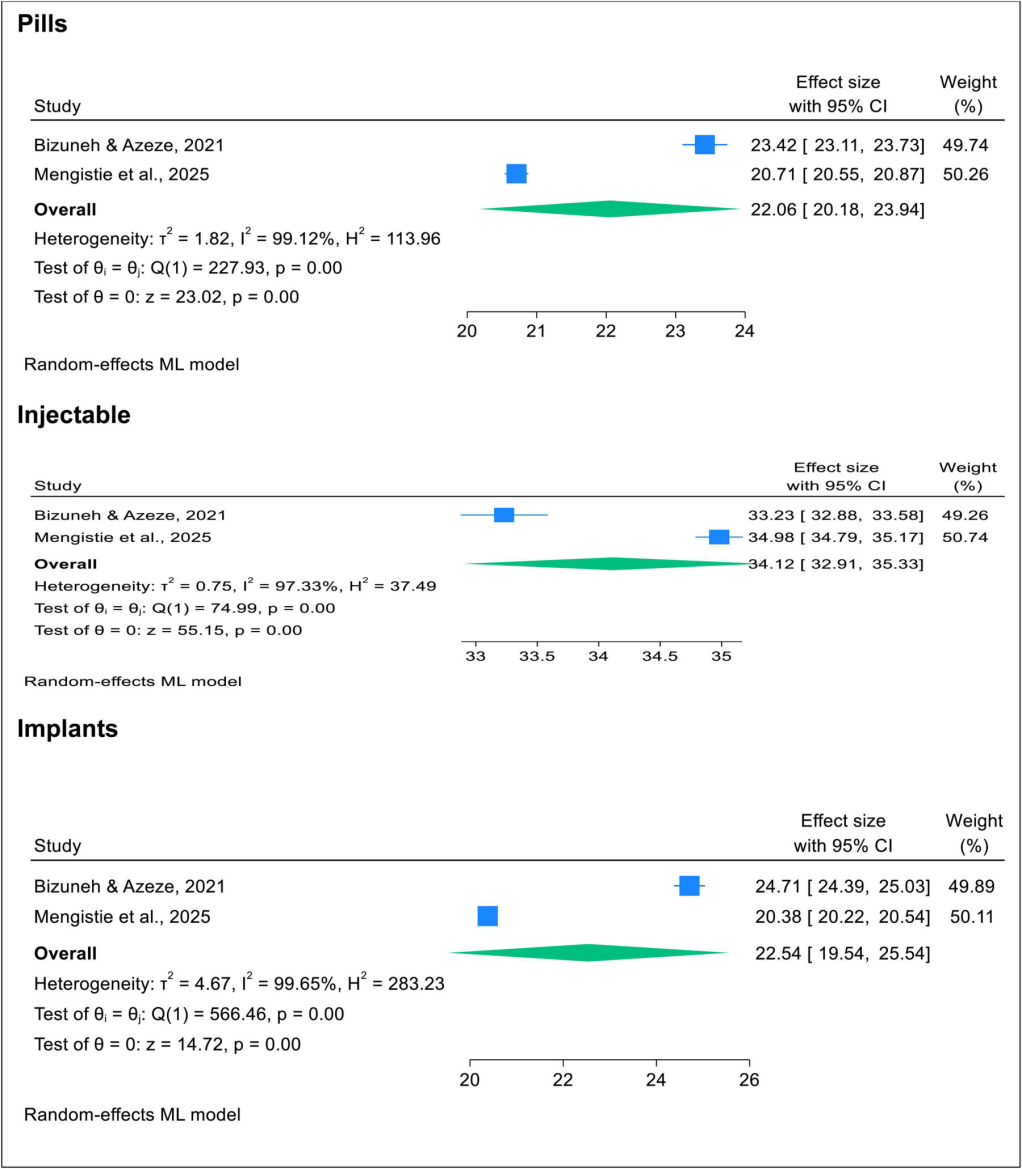

### Pooled key determinants of post-abortion family planning (FP) utilization

This umbrella review synthesized evidence to identify the key determinants of post-abortion family planning (FP) utilization among women of reproductive age in Africa. The analysis revealed that married women were significantly more likely to use FP services (AOR: 2.64; 95% CI: 2.09–3.19), as were younger women aged 15–24 years (AOR: 5.87; 95% CI: 2.44 –9.30). Educational attainment was positively associated with FP uptake (AOR: 2.22; 95% CI: 1.54–2.90), while receiving post-abortion FP counseling substantially increased utilization (AOR: 3.98; 95% CI: 3.31–4.65). Prior use of FP (AOR: 4.30; 95% CI: 2.91–5.70), history of abortion (AOR: 2.10; 95% CI: 1.79–2.42), unintended pregnancy (AOR: 4.06; 95% CI: 2.70–5.41), and contraceptive knowledge (AOR: 2.58; 95% CI: 2.19–2.97) were also identified as significant predictors. Despite the high heterogeneity across studies (*I*^2^ > 99%), the findings consistently underscore the critical role of education, counseling, and reproductive history in shaping post-abortion FP utilization. Further details on the identified factors for FP utilization are provided in Supplementary File 3.2 and Table 4.

**Table 4:** Pooled key determinants of post-abortion family planning (FP) utilization among women of reproductive age in 2025.

Author (reference)	Identified determinant	Pooled AOR	95% CI	Heterogeneity	No. of studies
Bizuneh and Azeze (15), Beyene et al. (16), Mengistie et al. (26)	Marital status (married)	2.64	2.09–3.19	*I*^2^ = 99.96%, *P* = 0.00	3
Bizuneh and Azeze (15), Mengistie et al. (26)	Maternal age (15–24 years)	5.87	2.44–9.30	*I*^2^ = 99.88%, *P* = 0.00	2
Bizuneh and Azeze (15), Beyene et al. (16), Mengistie et al. (26)	Maternal educational status	2.22	1.54–2.90	*I*^2^ = 99.99%, *P* = 0.00	3
Bizuneh and Azeze (15), Beyene et al. (16), Dagnew and Asresie (18), Cherie et al. (25), Mengistie et al. (26), Wake et al. (27)	Post-abortion care, FP counseling	3.98	3.31–4.65	*I*^2^ = 99.96%, *P* = 0.00	6
Bizuneh and Azeze (15), Beyene et al. (16), Dagnew and Asresie (18), Cherie et al. (25), Mengistie et al. (26), Wake et al. (27)	Ever used family planning	4.30	2.91–5.70	*I*^2^ = 99.99%, *P* = 0.00	6
Bizuneh and Azeze (15), Mengistie et al. (26)	History of abortion	2.10	1.79–2.42	*I*^2^ = 99.95%, *P* = 0.00	2
Dagnew and Asresie (18), Mengistie et al. (26)	Unintended pregnancy	4.06	2.70–5.41	*I*^2^ = 99.98%, *P* = 0.00	2
Dagnew and Asresie (18), Mengistie et al. (26)	Contraceptive knowledge	2.58	2.19–2.97	*I*^2^ = 99.92%, *P* = 0.00	2

### Post-abortion family planning (FP) utilization interventions

Recent findings from previous meta-analyses of prospective or randomized controlled studies on post-abortion family planning (FP) interventions highlight several key insights. For instance, a study by Buckingham et al. found that immediate postpartum use of long-acting reversible contraceptives (LARCs) is generally acceptable among adolescents, particularly when side effects are minimal and decision-making autonomy is respected. However, evidence on post-abortion LARC use among adolescents remains limited (28). Similarly, Roe and Bartz concluded that most reversible contraceptive methods are safe following surgical abortion, although the optimal timing for initiation varies in cases of medical abortion. They emphasized that abortion care presents a critical opportunity to offer access to long-acting contraceptive methods (30).

Further, Schmidt-Hansen et al. reported that early administration of LARCs during medical abortion enhances user satisfaction and reduces the risk of unintended pregnancies. Early insertion of intrauterine devices (IUDs) was associated with increased uptake and continued use, supporting equitable, person-centered care (33).

Another study by Sedlecky and Stanković found that immediate post-abortion initiation of reliable contraception, combined with adolescent-centered counseling, effectively prevents repeat pregnancies. While LARC methods are safe, maintaining long-term adherence may be challenging (34). In China, Wang et al. observed that post-abortion care significantly improved reproductive outcomes, including increased contraceptive use and reduced repeat abortions, although they noted the need for high-quality research to optimize service delivery (36).

Rogers and Dantas highlighted that access to contraception and SRH information following abortion is often hindered by limited education and negative provider attitudes. They emphasized the importance of culturally safe counseling to improve contraceptive acceptance (31). Similarly, Rossier et al. found that abortion disclosure within social networks is influenced by stigma and the desire for anonymity. Informal medical abortion use did not significantly affect disclosure patterns, underscoring the need for stigma-sensitive interventions to enhance access to care (32). Table 5 summarizes the key findings on the impacts of post-abortion family planning (FP) interventions and the significant conclusions reported by the authors.

**Table 5:** Summary of key findings on intervention impacts of post-abortion family planning (FP) utilization, highlighting the conclusions reported by each author in 2025.

Author (reference)	Review aim	Search strategy	No. of included studies	Study setting	Sample size	Conclusion	Quality MSTAR-2
Buckingham et al. (28)	This review explored adolescents' attitudes, experiences, and decision-making factors regarding immediate postpartum and post-abortion long-acting reversible contraception use and discontinuation	MEDLINE, E-pub Ahead of Print, Embase + Emcare, and PsycInfo via Ovid, CINAHL, and personal reference libraries	10	Globally	16,482	This review found that immediate postpartum long-acting reversible contraceptive (LARC) use is generally acceptable to adolescents, especially when side effects are minimal and they have autonomy in decision-making. However, research specifically focused on adolescents is limited, and evidence regarding post-abortion LARC use is scarce. Further studies are needed to explore adolescents' experiences and promote person-centered contraceptive care after pregnancy	12
Roe and Bartz (30)	This review seeks to provide up-to-date recommendations about the provision and timing of contraception after surgical and medical abortion					This review emphasizes collaborative contraceptive counseling during abortion care. Most reversible methods are safe following surgical abortion, barring complications. After medical abortion, timing varies: implants are immediate, hormonal methods follow soon after, and IUDs after completion. Research is needed on the timing of depot medroxyprogesterone acetate. Abortion care offers a key opportunity for contraceptive access, especially to long-acting reversible methods	11
Schmidt-Hansen et al. (33)	This review examined whether early administration of LARCs during medical abortion affects abortion efficacy and assessed the safety of intrauterine device insertion following expulsion of conception products	Embase; Ovid MEDLINE(R) including Daily and Epub Ahead of Print, In-Process and Cochrane Library; Cinahl Plus	28	Globally	6,709,643	This review explored the impact of early LARC administration during medical abortion and the safety of IUD insertion after expulsion. Simultaneous use of mifepristone and LARCs, such as implants or injectables, improves patient satisfaction and reduces unintended pregnancies. Early IUC insertion after abortion increases uptake and continuation, with some variation by gestational age. Despite varying evidence quality, early LARC provision is recommended to enhance access, especially for vulnerable women, and universal early insertion promotes equitable, person-centered contraceptive care	14
Sedlecky and Stanković (34)	This study reviewed the recommended methods and counseling for effective prevention of repeat pregnancies in adolescents	Medline, Science Direct, Google, and Popline, along with expert opinions		Globally		This review highlights effective strategies to prevent repeat adolescent pregnancies, emphasizing tailored, non-directive contraceptive counseling, immediate post-abortion initiation of reliable methods, and ongoing support. Adolescents are most motivated right after abortion, with ovulation resuming in three weeks and sexual activity often within two. Long-acting reversible contraceptives (LARCs) are safe and effective, although their long-term use may be challenging. Adolescent-centered care enhances continuity and supports sustained contraceptive use, helping reduce repeat pregnancies and improve reproductive outcomes	12
Ferreira et al. (29)	This study performed a systematic review of the effectiveness of contraceptive counseling in women undergoing an abortion and its impact on the acceptance and use of contraceptive methods	LILACS, SCIELO, MEDLINE, PubMed, and Cochrane Library databases from 1997 to 2007	3	Globally	694	This systematic review found no significant effect of post-abortion contraceptive counseling on method uptake. Despite variability across trials, no intervention-related differences were observed. The findings may not be generalizable to developing countries, where sociocultural and economic factors influence outcomes. Further research is needed to develop context-specific, effective strategies	12
Wang et al. (36)	This study aimed to evaluate the effectiveness of post-abortion care services in Chinese women who have undergone induced abortion	PubMed, EMBASE, Web of Science, WHO ICTRP, and Chinese electronic databases (CNKI and Wanfang)	42	China	70,126	A review of 42 randomized controlled trials in China involving over 70,000 participants found that post-abortion care significantly improved reproductive outcomes. It increased contraceptive use, reduced repeat abortions, improved follow-up rates, and enhanced satisfaction. Despite varying evidence quality, post-abortion care supports fertility protection and contraceptive continuation	15
						Further high-quality research is needed to optimize service delivery and ensure consistent care	
Stewart et al. (12)	This systematic review and meta-analysis assessed whether enhanced peri-abortion contraceptive counseling had an effect on subsequent unplanned pregnancies and the uptake and continuation of contraceptive methods	MEDLINE, EMBASE, and the Cochrane Library. Only randomized controlled trials (RCTs) involving enhanced pre- and post-abortion contraceptive counseling were utilized	6	Globally		This systematic review and meta-analysis evaluated the impact of enhanced peri-abortion contraceptive counseling on unplanned pregnancies and contraceptive use. It found no significant evidence supporting its effectiveness in reducing repeat pregnancies or improving uptake and continuation of LARC. Limitations included small sample sizes, short follow-up periods, and high heterogeneity. These factors may have influenced outcomes. Future research should focus on larger, multicenter trials with longer follow-up periods and explore interventions that support sustained use of effective contraception	16
Tripney et al. (35)	This study examined the impact of post-abortion family planning counseling and services in low-income countries	Published and unpublished literature was utilized. Search conducted in two phases, involving ten electronic bibliographic databases/specialist registers and websites of nine organizations	15	Globally		This systematic review assessed post-abortion family planning counseling and services in low-income countries. Due to poor-quality evaluations and limited outcome data, evidence was inconclusive regarding impacts on maternal mortality, morbidity, repeat abortions, or unplanned pregnancies. Some women accepted modern contraceptives, especially oral pills and injectables, though reporting was inconsistent. The review emphasized the need for improved data collection and evaluation. Emerging research, supported by NGO–government collaborations, shows promise, with future studies focusing on contraceptive use and repeat abortion prevention	15
Rogers and Dantas (31)	This systematic literature review documented, analyzed, and critiqued the accessibility of contraception and sexual and reproductive health (SRH) information for women living in low- and middle-income countries who have undergone medical or surgical abortion	Ovid (MEDLINE), ProQuest, Science Direct, Web of Science, PUBMED, and CINAHL databases	9	Globally		This systematic review examined access to contraception and sexual and reproductive health (SRH) information for women in low- and middle-income countries after abortion. Key barriers include limited SRH education and negative provider attitudes. When women are offered a wide range of contraceptive options and culturally safe counseling, acceptance of post-abortion contraception increases. The review calls for clearer definitions of “counseling” and “cultural safety” to improve care quality and ensure comprehensive, supportive post-abortion services	14
Rossier et al. (32)	This study identified the degree of disclosure to social network members in restrictive LMICs and explored the differences between women obtaining an informal medical abortion and other abortion seekers	Pubmed, POPLINE, AIMS, LILACS, IMSEAR, and WPRIM databases	79	LMICs		This systematic review of 79 studies across 33 low- and middle-income countries examined abortion disclosure within social networks under restrictive conditions. Disclosure varied by country type, with most studies in Type 3 (non-anonymous access, high stigma). Disclosure increased with reduced stigma and greater anonymity, but informal medical abortion use did not affect disclosure. No studies fit Type 4. Findings underscore the need for stigma-sensitive, network-based interventions to improve access to safe abortion information and services	15

## Discussion

This umbrella review of systematic reviews and meta-analyses found that the pooled prevalence of post-abortion family planning utilization among women of reproductive age in Africa was 62.82% (95% CI: 59.24%–66.40%), with Ethiopia showing a higher rate of 69.31% compared to 60.29% in other African countries. Injectables were the most commonly used method, followed by pills and implants.

This study identified key pooled determinants that influence post-abortion family planning use in Africa, including being married, younger age (15–24), higher educational attainment, prior contraceptive use, abortion history, unintended pregnancy, and receiving counseling. These determinants suggest that targeted education, counseling, and support for young, unmarried, and less-informed women could improve utilization rates. Furthermore, this umbrella review found that previous post-abortion family planning interventions improve reproductive outcomes by increasing contraceptive use and reducing repeat abortions. All included studies underwent thorough quality assessments, consistently receiving AMSTAR-2 ratings between 14 and 16, confirming strong methodological rigor and reliability across the findings.

This umbrella review of systematic reviews and meta-analyses found that the pooled prevalence of post-abortion family planning utilization among women of reproductive age in Africa was 62.82% (95% CI: 59.24%–66.40%) (15, 16, 18, 25–27) with Ethiopia showing a higher rate of 69.31% (16, 25, 27) compared to 60.29% in other African countries (15, 18, 26). This is further supported by another study, which emphasized the critical role of PAFP in reducing unintended pregnancies and unsafe abortions in Eastern Africa, reporting utilization rates between 53.7% and 67.86% (14, 15). Although these figures fall short of WHO targets, they reflect meaningful progress in preventing unintended pregnancies and repeat abortions. The higher PAFP uptake observed in Ethiopia may reflect better access to nationally integrated abortion and post-abortion family planning services, increased awareness, and more effective service delivery systems (38). However, the fact that nearly half of the women in some regions still do not use PAFP underscores the urgent need for improved counseling, education, and culturally tailored interventions to address regional disparities and enhance family planning services across the continent (9, 10).

This study identified several pooled key determinants influencing PAFP utilization in Africa, including marital status, younger age (15–24), higher education, prior contraceptive use, abortion history, unintended pregnancy, and receipt of counseling (15, 16, 18, 25–27). These findings align with previous reviews, which consistently highlight education, marital status, and counseling as major factors influencing PAFP uptake (15–18). These determinants suggest that targeted education, counseling, and support for young, unmarried, and less-informed women could improve utilization rates.

Furthermore, this umbrella review found that previous post-abortion family planning (PAFP) interventions significantly improved reproductive outcomes by increasing contraceptive use and reducing repeat abortions. For example, immediate initiation of contraception and adolescent-focused counseling have proven effective, although maintaining long-term adherence to long-acting reversible contraceptives (LARCs) remains challenging. Persistent barriers such as limited education, provider bias, and stigma continue to restrict access to care and discourage open disclosure. Addressing these issues requires culturally sensitive, stigma-aware counseling approaches to enhance service delivery and promote acceptance of family planning interventions (31–34, 36). These findings are further supported by the 2022 WHO Abortion Care Guideline, which frames safe abortion and PAFP as health and human rights priorities. The guideline advocates for immediate post-abortion contraception and the expansion of provider roles, including community health workers and pharmacists, to reduce repeat unintended pregnancies and improve maternal health through accessible, high-quality care (9, 10).

### Strengths and limitations of the umbrella review

#### Strengths of the study

This umbrella review provides a robust and comprehensive synthesis of existing systematic reviews and meta-analyses on post-abortion family planning (PAFP) utilization among women of reproductive age in Africa. By consolidating fragmented evidence, it offers clearer insights into prevalence patterns and key determinants that are critical for informing health policy and improving service delivery. This study applied rigorous inclusion criteria and quality appraisal methods using the AMSTAR-2 tool, with scores ranging from 14 to 16, thereby ensuring methodological integrity. Its regional focus enhances contextual relevance, addressing the unique challenges and opportunities within African health systems. Furthermore, the review identifies research gaps and offers policy-relevant, evidence-based recommendations aimed at promoting reproductive health equity in low-resource settings. The inclusion of both published and unpublished studies strengthens the comprehensiveness of the evidence base, while the exclusion of low-quality and non-systematic literature ensures the reliability and clarity of the findings.

### Limitations

While this umbrella review offers a comprehensive synthesis of systematic reviews and meta-analyses on post-abortion family planning (PAFP) in Africa, several limitations should be acknowledged. First, the findings are influenced by the quality of the included reviews, which may vary in methodological rigor. Despite efforts to minimize bias, publication bias remains a concern, as studies reporting significant results are more likely to be published. Second, variability in the definitions and measurements of PAFP across studies may affect the comparability and interpretation of pooled estimates. Third, the exclusion of non-English publications and qualitative studies may limit the depth and contextual richness of the findings, particularly in understanding cultural and systemic barriers to PAFP uptake. Finally, it is important to note that self-managed abortions are often underreported or undetected in facility-based studies, which may lead to underestimation of post-abortion family planning needs. These limitations suggest the need for more inclusive and methodologically diverse research to fully capture the complexity of PAFP utilization in African settings.

### Implications for public health and policy

The findings of this umbrella review underscore the urgent need for targeted policy interventions to improve post-abortion family planning (PAFP) utilization across Africa.

First, integrating PAFP counseling into routine post-abortion care is essential to ensure that women receive timely, informed, and supportive guidance on contraceptive options. This integration should be standardized across all health facilities and embedded within national reproductive health protocols.

Second, targeted interventions are needed for vulnerable groups, particularly younger women and those with low educational attainment, who face greater barriers to accessing family planning services. Youth-friendly and culturally sensitive approaches should be prioritized to address these disparities.

Third, strengthening health systems is critical to reducing regional inequalities in PAFP access. This includes improving service delivery infrastructure, training healthcare providers, and expanding outreach programs in underserved areas. By addressing these policy gaps, countries can move closer to achieving reproductive health equity and reducing the incidence of unintended pregnancies and repeat abortions.

Strengthening health systems and community-based delivery of culturally sensitive, rights-based family planning services is essential to reduce repeat abortions and improve maternal health outcomes across the continent.

## Conclusion and recommendations

This umbrella review found that the pooled prevalence of post-abortion family planning (PAFP) utilization among women of reproductive age in Africa is 62.82%, with Ethiopia showing a higher uptake of 69.31%. Key determinants influencing PAFP use include younger age (15–24 years), being married, higher educational attainment, prior contraceptive use, history of abortion, unintended pregnancy, and receiving counseling. These factors highlight the need for targeted, equity-driven interventions.

To address the gaps in post-abortion family planning (PAFP) utilization across Africa, a coordinated, multisectoral strategy is essential. Governments, health systems, educational institutions, and community organizations must work in collaboration to strengthen reproductive health policies and ensure universal access to post-abortion care. Key actions include the following:

Integrating PAFP counseling into all levels of post-abortion care to support informed decision-making and continuity of contraceptive use;

Expanding access to a diverse range of contraceptive methods, particularly LARCs, to promote choice and reduce repeat abortions;

Targeting vulnerable populations, especially younger women and those with limited education, through youth-friendly services, community outreach, and tailored communication strategies; and

Strengthening health systems, especially in underserved regions, by investing in provider training, infrastructure, and equitable service delivery.

These measures will promote reproductive health equity, reduce unintended pregnancies, and improve maternal health outcomes across the continent.

Future research should explore the longitudinal outcomes of PAFP interventions, assess the impact of culturally tailored counseling strategies, and incorporate qualitative studies to better understand contextual barriers. Such efforts will support evidence-based policymaking and promote reproductive health equity across Africa.

## Data Availability

The original contributions presented in the study are included in the article/Supplementary Material, further inquiries can be directed to the corresponding author.
