# Supplementary material for: Evidence and determinants of post-abortion family planning utilization among women of reproductive age in Africa: an umbrella review
Source: Front Reprod Health. 2025 Nov 13;7:1687886. doi: 10.3389/frph.2025.1687886 (PMC12657429; doi:10.3389/frph.2025.1687886)
Supplement: Supplementary file 1 [file Table1.docx]

**S1: Search strategy and lists of included and excluded studies for Post-Abortion history and search details**

| **Search** | **Actions** | **Details** | **Query** | **Results** | **Time** |
| --- | --- | --- | --- | --- | --- |
| #33 |  |  | Search: **Similar articles for PMID: 40495240** Filters: **Full text, Meta-Analysis, Systematic Review, English, Humans** | [47](https://pubmed.ncbi.nlm.nih.gov/?filter=simsearch3.fft&filter=pubt.meta-analysis&filter=pubt.systematicreview&filter=lang.english&filter=hum_ani.humans&linkname=pubmed_pubmed&from_uid=40495240&sort=relevance) | 02:31:43 |
| #46 |  |  | Search: **((((((post-abortion contraception) OR (post-abortion family planning )) OR ("Contraception"[Mesh] OR "Contraception, Postcoital"[Mesh])) OR ("Contraceptive Agents"[Mesh] OR "Contraceptive Agents" [Pharmacological Action] OR "Contraceptive Devices"[Mesh] OR "Contraceptive Agents, Hormonal"[Mesh])) OR ("Family Planning Services"[Mesh])) AND "Epidemiologic Factors"[Mesh])) AND ("Prevalence"[Mesh] OR "Epidemiology"[Mesh] OR "Contraceptive Prevalence Surveys"[Mesh] OR "epidemiology" [Subheading])** Filters: **Full text, Meta-Analysis, Systematic Review, English, Humans, Female** | [97](https://pubmed.ncbi.nlm.nih.gov/?term=%28%28%28%28%28%28post-abortion+contraception%29+OR+%28post-abortion+foamily+planning+%29%29+OR+%28%22Contraception%22%5BMesh%5D+OR+%22Contraception%2C+Postcoital%22%5BMesh%5D%29%29+OR+%28%22Contraceptive+Agents%22%5BMesh%5D+OR+%22Contraceptive+Agents%22+%5BPharmacological+Action%5D+OR+%22Contraceptive+Devices%22%5BMesh%5D+OR+%22Contraceptive+Agents%2C+Hormonal%22%5BMesh%5D%29%29+OR+%28%22Family+Planning+Services%22%5BMesh%5D%29%29+AND+%22Epidemiologic+Factors%22%5BMesh%5D%29%29+AND+%28%22Prevalence%22%5BMesh%5D+OR+%22Epidemiology%22%5BMesh%5D+OR+%22Contraceptive+Prevalence+Surveys%22%5BMesh%5D+OR+%22epidemiology%22+%5BSubheading%5D%29&filter=simsearch3.fft&filter=pubt.meta-analysis&filter=pubt.systematicreview&filter=lang.english&filter=hum_ani.humans&filter=sex.female&sort=relevance) | 02:29:22 |
| #45 |  |  | Search: **((((((post-abortion contraception) OR (post-abortion family planning )) OR ("Contraception"[Mesh] OR "Contraception, Postcoital"[Mesh])) OR ("Contraceptive Agents"[Mesh] OR "Contraceptive Agents" [Pharmacological Action] OR "Contraceptive Devices"[Mesh] OR "Contraceptive Agents, Hormonal"[Mesh])) OR ("Family Planning Services"[Mesh])) AND "Epidemiologic Factors"[Mesh])) AND ("Prevalence"[Mesh] OR "Epidemiology"[Mesh] OR "Contraceptive Prevalence Surveys"[Mesh] OR "epidemiology" [Subheading])** Filters: **Full text, Associated data, Meta-Analysis, Systematic Review, English, Humans, Female** | [20](https://pubmed.ncbi.nlm.nih.gov/?term=%28%28%28%28%28%28post-abortion+contraception%29+OR+%28post-abortion+foamily+planning+%29%29+OR+%28%22Contraception%22%5BMesh%5D+OR+%22Contraception%2C+Postcoital%22%5BMesh%5D%29%29+OR+%28%22Contraceptive+Agents%22%5BMesh%5D+OR+%22Contraceptive+Agents%22+%5BPharmacological+Action%5D+OR+%22Contraceptive+Devices%22%5BMesh%5D+OR+%22Contraceptive+Agents%2C+Hormonal%22%5BMesh%5D%29%29+OR+%28%22Family+Planning+Services%22%5BMesh%5D%29%29+AND++%22Epidemiologic+Factors%22%5BMesh%5D%29%29+AND+%28%22Prevalence%22%5BMesh%5D+OR+%22Epidemiology%22%5BMesh%5D+OR+%22Contraceptive+Prevalence+Surveys%22%5BMesh%5D+OR+%22epidemiology%22+%5BSubheading%5D%29&filter=simsearch3.fft&filter=articleattr.data&filter=pubt.meta-analysis&filter=pubt.systematicreview&filter=lang.english&filter=hum_ani.humans&filter=sex.female&sort=relevance) | 02:27:47 |
| #44 |  |  | Search: **((((((post-abortion contraception) OR (post-abortion family planning )) OR ("Contraception"[Mesh] OR "Contraception, Postcoital"[Mesh])) OR ("Contraceptive Agents"[Mesh] OR "Contraceptive Agents" [Pharmacological Action] OR "Contraceptive Devices"[Mesh] OR "Contraceptive Agents, Hormonal"[Mesh])) OR ("Family Planning Services"[Mesh])) OR ("Social Determinants of Health"[Mesh] OR "Epidemiologic Factors"[Mesh])) AND ("Prevalence"[Mesh] OR "Epidemiology"[Mesh] OR "Contraceptive Prevalence Surveys"[Mesh] OR "epidemiology" [Subheading])** Filters: **Full text, Associated data, Meta-Analysis, Systematic Review, English, Humans, Female** | [4,084](https://pubmed.ncbi.nlm.nih.gov/?term=%28%28%28%28%28%28post-abortion+contraception%29+OR+%28post-abortion+foamily+planning+%29%29+OR+%28%22Contraception%22%5BMesh%5D+OR+%22Contraception%2C+Postcoital%22%5BMesh%5D%29%29+OR+%28%22Contraceptive+Agents%22%5BMesh%5D+OR+%22Contraceptive+Agents%22+%5BPharmacological+Action%5D+OR+%22Contraceptive+Devices%22%5BMesh%5D+OR+%22Contraceptive+Agents%2C+Hormonal%22%5BMesh%5D%29%29+OR+%28%22Family+Planning+Services%22%5BMesh%5D%29%29+OR+%28%22Social+Determinants+of+Health%22%5BMesh%5D+OR+%22Epidemiologic+Factors%22%5BMesh%5D%29%29+AND+%28%22Prevalence%22%5BMesh%5D+OR+%22Epidemiology%22%5BMesh%5D+OR+%22Contraceptive+Prevalence+Surveys%22%5BMesh%5D+OR+%22epidemiology%22+%5BSubheading%5D%29&filter=simsearch3.fft&filter=articleattr.data&filter=pubt.meta-analysis&filter=pubt.systematicreview&filter=lang.english&filter=hum_ani.humans&filter=sex.female&sort=relevance) | 02:26:25 |
| #43 |  |  | Search: **((((((post-abortion contraception) OR (post-abortion family planning )) OR ("Contraception"[Mesh] OR "Contraception, Postcoital"[Mesh])) OR ("Contraceptive Agents"[Mesh] OR "Contraceptive Agents" [Pharmacological Action] OR "Contraceptive Devices"[Mesh] OR "Contraceptive Agents, Hormonal"[Mesh])) OR ("Family Planning Services"[Mesh])) OR ("Social Determinants of Health"[Mesh] OR "Epidemiologic Factors"[Mesh])) AND ("Prevalence"[Mesh] OR "Epidemiology"[Mesh] OR "Contraceptive Prevalence Surveys"[Mesh] OR "epidemiology" [Subheading])** Filters: **Full text, Associated data, Meta-Analysis, Systematic Review, English, Humans** | [8,847](https://pubmed.ncbi.nlm.nih.gov/?term=%28%28%28%28%28%28post-abortion+contraception%29+OR+%28post-abortion+foamily+planning+%29%29+OR+%28%22Contraception%22%5BMesh%5D+OR+%22Contraception%2C+Postcoital%22%5BMesh%5D%29%29+OR+%28%22Contraceptive+Agents%22%5BMesh%5D+OR+%22Contraceptive+Agents%22+%5BPharmacological+Action%5D+OR+%22Contraceptive+Devices%22%5BMesh%5D+OR+%22Contraceptive+Agents%2C+Hormonal%22%5BMesh%5D%29%29+OR+%28%22Family+Planning+Services%22%5BMesh%5D%29%29+OR+%28%22Social+Determinants+of+Health%22%5BMesh%5D+OR+%22Epidemiologic+Factors%22%5BMesh%5D%29%29+AND+%28%22Prevalence%22%5BMesh%5D+OR+%22Epidemiology%22%5BMesh%5D+OR+%22Contraceptive+Prevalence+Surveys%22%5BMesh%5D+OR+%22epidemiology%22+%5BSubheading%5D%29&filter=simsearch3.fft&filter=articleattr.data&filter=pubt.meta-analysis&filter=pubt.systematicreview&filter=lang.english&filter=hum_ani.humans&sort=relevance) | 02:26:12 |
| #42 |  |  | Search: **((((((post-abortion contraception) OR (post-abortion family planning )) OR ("Contraception"[Mesh] OR "Contraception, Postcoital"[Mesh])) OR ("Contraceptive Agents"[Mesh] OR "Contraceptive Agents" [Pharmacological Action] OR "Contraceptive Devices"[Mesh] OR "Contraceptive Agents, Hormonal"[Mesh])) OR ("Family Planning Services"[Mesh])) OR ("Social Determinants of Health"[Mesh] OR "Epidemiologic Factors"[Mesh])) AND ("Prevalence"[Mesh] OR "Epidemiology"[Mesh] OR "Contraceptive Prevalence Surveys"[Mesh] OR "epidemiology" [Subheading])** Filters: **Full text, Associated data, Meta-Analysis, Systematic Review, English** | [8,889](https://pubmed.ncbi.nlm.nih.gov/?term=%28%28%28%28%28%28post-abortion+contraception%29+OR+%28post-abortion+foamily+planning+%29%29+OR+%28%22Contraception%22%5BMesh%5D+OR+%22Contraception%2C+Postcoital%22%5BMesh%5D%29%29+OR+%28%22Contraceptive+Agents%22%5BMesh%5D+OR+%22Contraceptive+Agents%22+%5BPharmacological+Action%5D+OR+%22Contraceptive+Devices%22%5BMesh%5D+OR+%22Contraceptive+Agents%2C+Hormonal%22%5BMesh%5D%29%29+OR+%28%22Family+Planning+Services%22%5BMesh%5D%29%29+OR+%28%22Social+Determinants+of+Health%22%5BMesh%5D+OR+%22Epidemiologic+Factors%22%5BMesh%5D%29%29+AND+%28%22Prevalence%22%5BMesh%5D+OR+%22Epidemiology%22%5BMesh%5D+OR+%22Contraceptive+Prevalence+Surveys%22%5BMesh%5D+OR+%22epidemiology%22+%5BSubheading%5D%29&filter=simsearch3.fft&filter=articleattr.data&filter=pubt.meta-analysis&filter=pubt.systematicreview&filter=lang.english&sort=relevance) | 02:24:56 |
| #41 |  |  | Search: **((((((post-abortion contraception) OR (post-abortion family planning )) OR ("Contraception"[Mesh] OR "Contraception, Postcoital"[Mesh])) OR ("Contraceptive Agents"[Mesh] OR "Contraceptive Agents" [Pharmacological Action] OR "Contraceptive Devices"[Mesh] OR "Contraceptive Agents, Hormonal"[Mesh])) OR ("Family Planning Services"[Mesh])) OR ("Social Determinants of Health"[Mesh] OR "Epidemiologic Factors"[Mesh])) AND ("Prevalence"[Mesh] OR "Epidemiology"[Mesh] OR "Contraceptive Prevalence Surveys"[Mesh] OR "epidemiology" [Subheading])** Filters: **Full text, Associated data, Meta-Analysis, Systematic Review** | [9,051](https://pubmed.ncbi.nlm.nih.gov/?term=%28%28%28%28%28%28post-abortion+contraception%29+OR+%28post-abortion+foamily+planning+%29%29+OR+%28%22Contraception%22%5BMesh%5D+OR+%22Contraception%2C+Postcoital%22%5BMesh%5D%29%29+OR+%28%22Contraceptive+Agents%22%5BMesh%5D+OR+%22Contraceptive+Agents%22+%5BPharmacological+Action%5D+OR+%22Contraceptive+Devices%22%5BMesh%5D+OR+%22Contraceptive+Agents%2C+Hormonal%22%5BMesh%5D%29%29+OR+%28%22Family+Planning+Services%22%5BMesh%5D%29%29+OR+%28%22Social+Determinants+of+Health%22%5BMesh%5D+OR+%22Epidemiologic+Factors%22%5BMesh%5D%29%29+AND+%28%22Prevalence%22%5BMesh%5D+OR+%22Epidemiology%22%5BMesh%5D+OR+%22Contraceptive+Prevalence+Surveys%22%5BMesh%5D+OR+%22epidemiology%22+%5BSubheading%5D%29&filter=simsearch3.fft&filter=articleattr.data&filter=pubt.meta-analysis&filter=pubt.systematicreview&sort=relevance) | 02:24:48 |
| #40 |  |  | Search: **((((((post-abortion contraception) OR (post-abortion family planning )) OR ("Contraception"[Mesh] OR "Contraception, Postcoital"[Mesh])) OR ("Contraceptive Agents"[Mesh] OR "Contraceptive Agents" [Pharmacological Action] OR "Contraceptive Devices"[Mesh] OR "Contraceptive Agents, Hormonal"[Mesh])) OR ("Family Planning Services"[Mesh])) OR ("Social Determinants of Health"[Mesh] OR "Epidemiologic Factors"[Mesh])) AND ("Prevalence"[Mesh] OR "Epidemiology"[Mesh] OR "Contraceptive Prevalence Surveys"[Mesh] OR "epidemiology" [Subheading])** Filters: **Full text, Associated data, Systematic Review** | [5,791](https://pubmed.ncbi.nlm.nih.gov/?term=%28%28%28%28%28%28post-abortion+contraception%29+OR+%28post-abortion+foamily+planning+%29%29+OR+%28%22Contraception%22%5BMesh%5D+OR+%22Contraception%2C+Postcoital%22%5BMesh%5D%29%29+OR+%28%22Contraceptive+Agents%22%5BMesh%5D+OR+%22Contraceptive+Agents%22+%5BPharmacological+Action%5D+OR+%22Contraceptive+Devices%22%5BMesh%5D+OR+%22Contraceptive+Agents%2C+Hormonal%22%5BMesh%5D%29%29+OR+%28%22Family+Planning+Services%22%5BMesh%5D%29%29+OR+%28%22Social+Determinants+of+Health%22%5BMesh%5D+OR+%22Epidemiologic+Factors%22%5BMesh%5D%29%29+AND+%28%22Prevalence%22%5BMesh%5D+OR+%22Epidemiology%22%5BMesh%5D+OR+%22Contraceptive+Prevalence+Surveys%22%5BMesh%5D+OR+%22epidemiology%22+%5BSubheading%5D%29&filter=simsearch3.fft&filter=articleattr.data&filter=pubt.systematicreview&sort=relevance) | 02:24:39 |
| #39 |  |  | Search: **((((((post-abortion contraception) OR (post-abortion family planning )) OR ("Contraception"[Mesh] OR "Contraception, Postcoital"[Mesh])) OR ("Contraceptive Agents"[Mesh] OR "Contraceptive Agents" [Pharmacological Action] OR "Contraceptive Devices"[Mesh] OR "Contraceptive Agents, Hormonal"[Mesh])) OR ("Family Planning Services"[Mesh])) OR ("Social Determinants of Health"[Mesh] OR "Epidemiologic Factors"[Mesh])) AND ("Prevalence"[Mesh] OR "Epidemiology"[Mesh] OR "Contraceptive Prevalence Surveys"[Mesh] OR "epidemiology" [Subheading])** Filters: **Full text, Associated data** | [186,727](https://pubmed.ncbi.nlm.nih.gov/?term=%28%28%28%28%28%28post-abortion+contraception%29+OR+%28post-abortion+foamily+planning+%29%29+OR+%28%22Contraception%22%5BMesh%5D+OR+%22Contraception%2C+Postcoital%22%5BMesh%5D%29%29+OR+%28%22Contraceptive+Agents%22%5BMesh%5D+OR+%22Contraceptive+Agents%22+%5BPharmacological+Action%5D+OR+%22Contraceptive+Devices%22%5BMesh%5D+OR+%22Contraceptive+Agents%2C+Hormonal%22%5BMesh%5D%29%29+OR+%28%22Family+Planning+Services%22%5BMesh%5D%29%29+OR+%28%22Social+Determinants+of+Health%22%5BMesh%5D+OR+%22Epidemiologic+Factors%22%5BMesh%5D%29%29+AND+%28%22Prevalence%22%5BMesh%5D+OR+%22Epidemiology%22%5BMesh%5D+OR+%22Contraceptive+Prevalence+Surveys%22%5BMesh%5D+OR+%22epidemiology%22+%5BSubheading%5D%29&filter=simsearch3.fft&filter=articleattr.data&sort=relevance) | 02:24:31 |
| #38 |  |  | Search: **((((((post-abortion contraception) OR (post-abortion family planning )) OR ("Contraception"[Mesh] OR "Contraception, Postcoital"[Mesh])) OR ("Contraceptive Agents"[Mesh] OR "Contraceptive Agents" [Pharmacological Action] OR "Contraceptive Devices"[Mesh] OR "Contraceptive Agents, Hormonal"[Mesh])) OR ("Family Planning Services"[Mesh])) OR ("Social Determinants of Health"[Mesh] OR "Epidemiologic Factors"[Mesh])) AND ("Prevalence"[Mesh] OR "Epidemiology"[Mesh] OR "Contraceptive Prevalence Surveys"[Mesh] OR "epidemiology" [Subheading])** Filters: **Full text** | [732,940](https://pubmed.ncbi.nlm.nih.gov/?term=%28%28%28%28%28%28post-abortion+contraception%29+OR+%28post-abortion+foamily+planning+%29%29+OR+%28%22Contraception%22%5BMesh%5D+OR+%22Contraception%2C+Postcoital%22%5BMesh%5D%29%29+OR+%28%22Contraceptive+Agents%22%5BMesh%5D+OR+%22Contraceptive+Agents%22+%5BPharmacological+Action%5D+OR+%22Contraceptive+Devices%22%5BMesh%5D+OR+%22Contraceptive+Agents%2C+Hormonal%22%5BMesh%5D%29%29+OR+%28%22Family+Planning+Services%22%5BMesh%5D%29%29+OR+%28%22Social+Determinants+of+Health%22%5BMesh%5D+OR+%22Epidemiologic+Factors%22%5BMesh%5D%29%29+AND+%28%22Prevalence%22%5BMesh%5D+OR+%22Epidemiology%22%5BMesh%5D+OR+%22Contraceptive+Prevalence+Surveys%22%5BMesh%5D+OR+%22epidemiology%22+%5BSubheading%5D%29&filter=simsearch3.fft&sort=relevance) | 02:24:20 |
| #37 |  |  | Search: **((((((post-abortion contraception) OR (post-abortion family planning )) OR ("Contraception"[Mesh] OR "Contraception, Postcoital"[Mesh])) OR ("Contraceptive Agents"[Mesh] OR "Contraceptive Agents" [Pharmacological Action] OR "Contraceptive Devices"[Mesh] OR "Contraceptive Agents, Hormonal"[Mesh])) OR ("Family Planning Services"[Mesh])) OR ("Social Determinants of Health"[Mesh] OR "Epidemiologic Factors"[Mesh])) AND ("Prevalence"[Mesh] OR "Epidemiology"[Mesh] OR "Contraceptive Prevalence Surveys"[Mesh] OR "epidemiology" [Subheading])** | [849,989](https://pubmed.ncbi.nlm.nih.gov/?term=%28%28%28%28%28%28post-abortion+contraception%29+OR++%28post-abortion+foamily+planning+%29%29+OR+%28%22Contraception%22%5BMesh%5D+OR+%22Contraception%2C+Postcoital%22%5BMesh%5D%29%29+OR+%28%22Contraceptive+Agents%22%5BMesh%5D+OR+%22Contraceptive+Agents%22+%5BPharmacological+Action%5D+OR+%22Contraceptive+Devices%22%5BMesh%5D+OR+%22Contraceptive+Agents%2C+Hormonal%22%5BMesh%5D%29%29+OR+%28%22Family+Planning+Services%22%5BMesh%5D%29%29+OR+%28%22Social+Determinants+of+Health%22%5BMesh%5D+OR+%22Epidemiologic+Factors%22%5BMesh%5D%29%29+AND+%28%22Prevalence%22%5BMesh%5D+OR+%22Epidemiology%22%5BMesh%5D+OR+%22Contraceptive+Prevalence+Surveys%22%5BMesh%5D+OR+%22epidemiology%22+%5BSubheading%5D%29&sort=) | 02:23:43 |
| #35 |  |  | Search: **post-abortion contraception** | [497](https://pubmed.ncbi.nlm.nih.gov/?term=post-abortion+contraception&sort=relevance) | 02:17:55 |
| #26 |  |  | Search: **"Family Planning Services"[Mesh]** Sort by: **Most Recent** | [27,190](https://pubmed.ncbi.nlm.nih.gov/?sort=date&term=%22Family+Planning+Services%22%5BMesh%5D) | 01:55:04 |
| #25 |  |  | Search: **"Contraceptive Agents"[Mesh] OR "Contraceptive Agents" [Pharmacological Action] OR "Contraceptive Devices"[Mesh] OR "Contraceptive Agents, Hormonal"[Mesh]** Sort by: **Most Recent** | [76,645](https://pubmed.ncbi.nlm.nih.gov/?sort=date&term=%22Contraceptive+Agents%22%5BMesh%5D+OR+%22Contraceptive+Agents%22+%5BPharmacological+Action%5D+OR+%22Contraceptive+Devices%22%5BMesh%5D+OR++%22Contraceptive+Agents%2C+Hormonal%22%5BMesh%5D) | 01:53:17 |
| #24 |  |  | Search: **"Social Determinants of Health"[Mesh] OR "Epidemiologic Factors"[Mesh]** Sort by: **Most Recent** | [1,855,524](https://pubmed.ncbi.nlm.nih.gov/?sort=date&term=%22Social+Determinants+of+Health%22%5BMesh%5D+OR++%22Epidemiologic+Factors%22%5BMesh%5D) | 01:48:45 |
| #23 |  |  | Search: **"Prevalence"[Mesh] OR "Epidemiology"[Mesh] OR "Contraceptive Prevalence Surveys"[Mesh] OR "epidemiology" [Subheading]** Sort by: **Most Recent** | [2,991,016](https://pubmed.ncbi.nlm.nih.gov/?sort=date&term=%22Prevalence%22%5BMesh%5D+OR+%22Epidemiology%22%5BMesh%5D+OR+%22Contraceptive+Prevalence+Surveys%22%5BMesh%5D+OR++%22epidemiology%22+%5BSubheading%5D) | 01:47:54 |
| #22 |  |  | Search: **"Abortion, Induced"[Mesh] OR "Abortion Applicants"[Mesh]** Sort by: **Most Recent** | [44,542](https://pubmed.ncbi.nlm.nih.gov/?sort=date&term=%22Abortion%2C+Induced%22%5BMesh%5D+OR++%22Abortion+Applicants%22%5BMesh%5D) | 01:46:05 |
